# Atomic Layer Deposition Derived Zirconia Coatings on Ni‐Rich Cathodes in Solid‐State Batteries: Correlation Between Surface Constitution and Cycling Performance

**DOI:** 10.1002/smsc.202200073

**Published:** 2022-12-11

**Authors:** David Kitsche, Yushu Tang, Hendrik Hemmelmann, Felix Walther, Matteo Bianchini, Aleksandr Kondrakov, Jürgen Janek, Torsten Brezesinski

**Affiliations:** ^1^ Battery and Electrochemistry Laboratory (BELLA) Institute of Nanotechnology Karlsruhe Institute of Technology (KIT) Hermann-von-Helmholtz-Platz 1 76344 Eggenstein-Leopoldshafen Germany; ^2^ Institute of Nanotechnology Karlsruhe Institute of Technology (KIT) Hermann-von-Helmholtz-Platz 1 76344 Eggenstein-Leopoldshafen Germany; ^3^ Institute of Physical Chemistry & Center for Materials Research (ZfM/LaMa) Justus-Liebig-University Giessen Heinrich-Buff-Ring 17 35392 Giessen Germany; ^4^ BASF SE Carl-Bosch-Strasse 38 67056 Ludwigshafen am Rhein Germany

**Keywords:** atomic layer deposition, cathode materials, electro-chemo-mechanical degradation, solid-state batteries, surface characterization

## Abstract

Protective coatings are required to address interfacial incompatibility issues in composite cathodes made from Ni‐rich layered oxides and lithium thiophosphate solid electrolytes (SEs), one of the most promising combinations of materials for high energy and power density solid‐state battery (SSB) applications. Herein, the preparation of conformal ZrO_2_ nanocoatings on a LiNi_0.85_Co_0.10_Mn_0.05_O_2_ (NCM85) cathode‐active material (CAM) by atomic layer deposition (ALD) is reported and the structural and chemical evolution of the modified NCM85 upon heat treatment—a post‐processing step often required to boost battery performance—is investigated. The coating properties are shown to have a strong effect on the cyclability of high‐loading SSB cells. After mild annealing (≈400 °C), the CAM delivers high specific capacities (≈200 mAh g^−1^ at C/10) and exhibits improved rate capability (≈125 mAh g^−1^ at 1C) and stability (≈78% capacity retention after 200 cycles at 0.5C), enabled by effective surface passivation. In contrast, annealing temperatures above 500 °C lead to the formation of an insulating interphase that negatively affects the cycling performance. The results of this study demonstrate that the preparation conditions for a given SE/CAM combination need to be tailored carefully and ALD is a powerful surface‐engineering technique toward this goal.

## Graphical abstract

The secondary particles of the cathode material LiNi_0.85_Co_0.10_Mn_0.05_O_2_ are conformally coated with ZrO_2_ nanoshells making use of the unique capabilities of atomic layer deposition (ALD). The multifaceted evolution of the surface structure upon post‐heat treatment and its effect on the cycling performance of thiophosphate‐based solid‐state batteries are studied systematically.

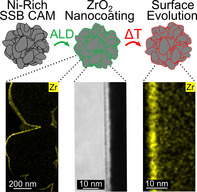

## 1 Introduction

Solid‐state batteries (SSBs) are the focus of research and public interest, especially because of the demand for safe electric vehicles with higher ranges and faster charging.^[^ 1, 2, 3 ^]^ This calls for materials enabling high energy densities and high (effective) ionic conductivities. When it comes to bulk‐type SSBs, the combination of thiophosphate (sulfide) solid electrolytes (SEs) and Ni‐rich layered oxide cathode‐active materials (CAMs), such as LiNi_*x*_Co_*y*_Mn_*z*_O_2_ (commonly referred to as NMC or NCM), is among those with the best chance of meeting these requirements. The main reasons for this are the unmatched room‐temperature ionic conductivities of lithium thiophosphates^[^ ^4^ ^]^ and the high specific capacities and half‐cell potentials of the technologically mature Ni‐rich NCM CAMs.^[^ ^5^ ^]^ Apart from the challenge of realizing the practical use of lithium‐metal or silicon anodes, a key requirement is the successful engineering of stable interfaces in SSBs.^[^ ^6^ ^]^ This applies especially to composite cathodes made from the aforementioned materials, which are typically operated at cell voltages above 4.0 V.

The degradation of lithium thiophosphates and potential mitigation strategies have been investigated in depth in the past decade.^[^ 6, 7, 8, 9 ^]^ Oxides, such as the prototype compound LiNbO_3_ and related stoichiometries, have been studied most extensively as coating materials, due to their stability and relative ease of preparation.^[^ 7, 10, 11, 12, 13, 14, 15 ^]^ For example, Zr‐based coatings in the form of ZrO_*x*_ or Li_*x*_Zr_*y*_O_*z*_ have been shown to be promising, with excellent cyclability of large‐format SSB cells demonstrated by Samsung.^[^ 16, 17, 18, 19, 20, 21 ^]^ These oxides were deposited onto the CAMs by various chemical and physical techniques.^[^ ^7^ ^]^ Among these, atomic layer deposition (ALD) stands out because of the unique capability of depositing conformal, (sub‐)nanometer films on complex substrates.^[^ 22, 23 ^]^ In liquid electrolyte‐based lithium‐ion batteries (LIBs), ALD coatings have already been proven to increase the cycle life.^[^ 24, 25, 26, 27, 28, 29 ^]^ The same approach can be used for application in the SSB context, but the coating chemistry might vary.^[^ 7, 29 ^]^ In fact, the deposition of several Li‐containing ternary oxides (attractive because of favorable ionic conductivities) has been reported.^[^ 30, 31, 32 ^]^ However, only a few studies have demonstrated a beneficial effect of ALD CAM coatings in bulk‐type SSB cells, e.g., for the SE/CAM combinations Li_3.15_Ge_0.15_P_0.85_S_4_ (LGPS)|Al_2_O_3_@LiCoO_2_ (LCO),^[^ ^33^ ^]^ Li_6_PS_5_Cl (LPSCl)|HfO_2_@NCM851005,^[^ ^34^ ^]^ LGPS|LiNbO_*x*_@NCM811,^[^ ^35^ ^]^ and LPSCl|LiO_*x*_‐ZrO_*x*_@LCO.^[^ ^20^ ^]^ The latter study revealed a superior performance of the Li‐containing coating over the binary counterpart ZrO_*x*_ in terms of rate capability and stability, which the authors attributed to improved ion migration across the SE|CAM interface. This is usually the rationale behind using ternary oxide coatings.^[^ ^7^ ^]^ Lithium incorporation into protective films can be achieved either directly by adding a lithium source along with the other precursors during synthesis^[^ 17, 19, 36 ^]^ or indirectly by an annealing step after coating, aiming at the reaction of residual lithium present on the CAM surface with the initially binary oxide coating.^[^ ^37^ ^]^ Considering that the ALD of Li‐based compounds is not trivial,^[^ ^38^ ^]^ it seems necessary to further explore the indirect route.

In the present work, we focus on ALD‐derived zirconia nanocoatings on LiNi_0.85_Co_0.10_Mn_0.05_O_2_ (hereafter referred to as ZrO_2_@NCM85) and systematically study the influence of post‐heat treatment on the CAM surface structure, composition, and morphology. In addition, we examine its effect on the cycling performance of the resulting ZrO_2_@NCM85 samples in pelletized SSB cells with an argyrodite LPSCl SE and a Li_4_Ti_5_O_12_ (LTO) composite anode. Post‐mortem investigations were also carried out to gain insights into the electro‐chemo‐mechanical degradation of the cathodes and to identify persisting stability issues.

## 2 Results and Discussion

**Figure 1:**
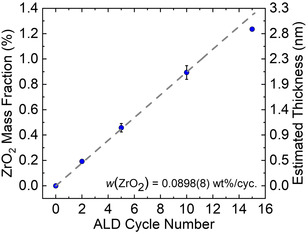
ZrO_2_ mass fraction and estimated film thickness on the NCM85 substrate depending on the number of tetrakis(ethylmethylamido)zirconium(IV) (TEMAZ)/O_3_ atomic layer deposition (ALD) cycles. The error bars indicate the standard deviation of measurements conducted on different batches of the ZrO_2_@NCM85. The gray dashed line is a linear fit to the data with a fixed *y*‐axis intercept at zero.

Prior to ALD, the NCM85 CAM was heated for 3 h in O_2_ flow at 750 °C, resulting in a carbonate content of ≈0.5 wt%.^[^ 34, 39 ^]^ This step helped to avoid too high levels of residual lithium, which has been shown to limit the performance both in LIB and SSB cells,^[^ 17, 40 ^]^ and further to improve the reliability of the coating process. Tetrakis(ethylmethylamido)zirconium(IV) (TEMAZ) and ozone were used to deposit ZrO_2_ onto the CAM secondary particles at a reactor temperature of 250 °C, an established procedure for the preparation of high‐quality zirconia films.^[^ ^41^ ^]^ Although ZrO_2_ ALD with H_2_O as a counter reactant is feasible, O_3_ was chosen based on previous cycling data obtained on HfO_2_‐coated NCM85 in analogous SSB cells.^[^ ^34^ ^]^ **Figure** **1** shows a plot of the mass fraction of ZrO_2_ determined by inductively coupled plasma‐optical emission spectroscopy (ICP‐OES) and the corresponding estimated film thickness on the CAM substrate as a function of the number of ALD cycles. The relationship was virtually linear, with a growth per cycle of ≈0.09 wt% ZrO_2_. Depositions on 2–8 g batches were found to follow the same trend, suggesting good saturation of the NCM85 surface by the pulsing sequence utilized in this work. The method is therefore suited for precisely tailoring the coating content/thickness. The thickness *t* can be estimated as follows: t=wm/ρA, where *A* and *m* represent the free surface area and total mass of the sample, respectively, and *ρ* and *w* are the density and mass fraction of the coating material. The growth rate amounted to ≈0.21 nm per ALD cycle. However, estimates using the Brunauer–Emmett–Teller (BET) surface area of the substrate, *A*_BET_  = 0.71 m^2^ g_NCM85_^−1^, and the crystallographic density of tetragonal ZrO_2_, *ρ* = 6.1 g cm^−3^, should be considered as lower bounds, since the actual coating density and area are probably somewhat lower.

Screening of the cycling performance in SSB cells revealed major improvements in capacity and rate capability for the as‐coated CAMs (after 5, 10, and 15 TEMAZ/O_3_ ALD cycles, see Figure S1, Supporting Information). While showing similar initial specific capacities, the 10 ALD cycle sample (≈0.9 wt% ZrO_2_) delivered larger reversible capacities in the later cycles, especially at higher rates. Because previous studies have shown that the post‐treatment has a strong effect on the performance of CAMs coated with binary oxides,^[^ 34, 37, 42 ^]^ we decided to investigate the impact of such a processing step in a systematic manner, with emphasis placed on the annealing temperature.

**Figure 2:**
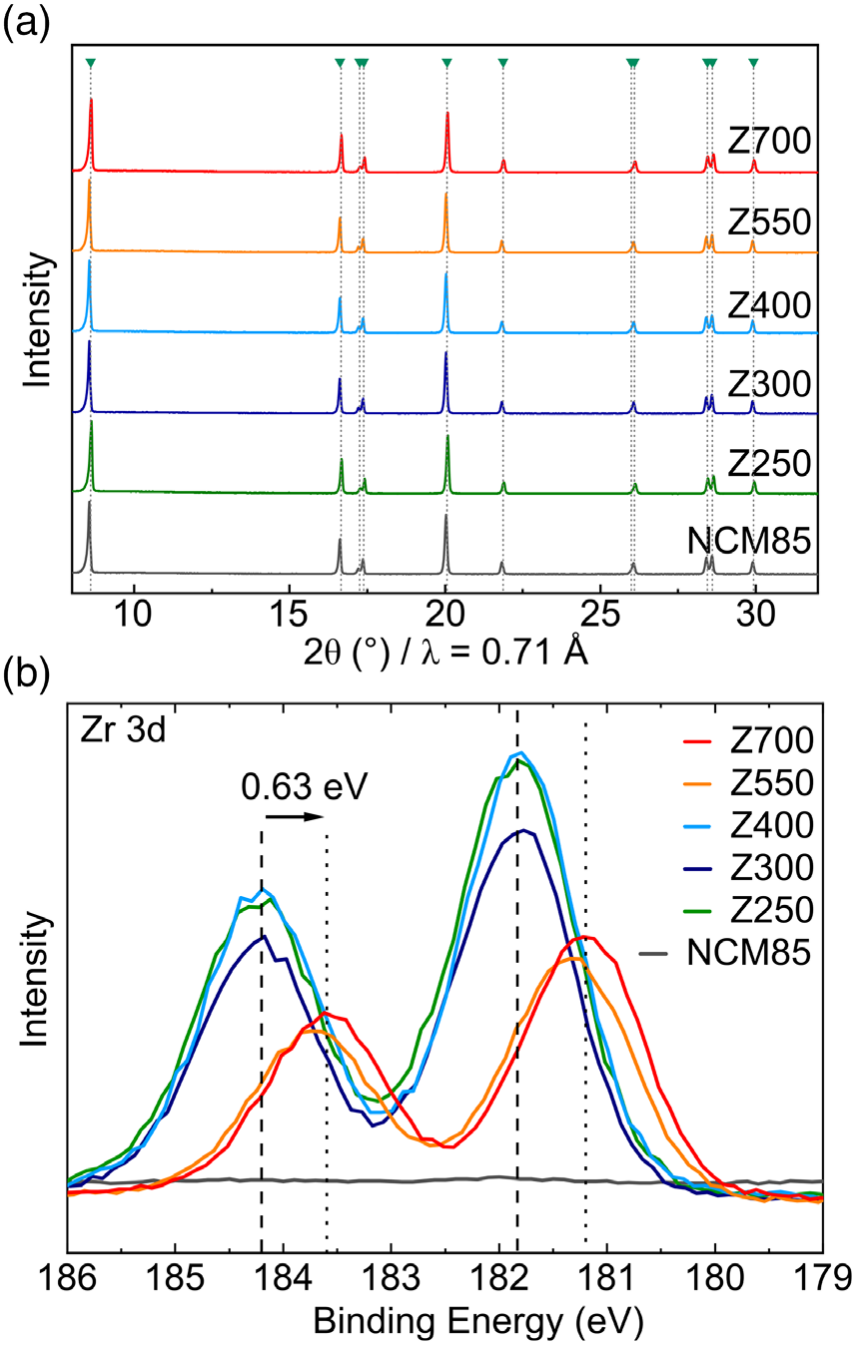
a) X‐ray diffraction (XRD) patterns of the uncoated NCM85 CAM and the ZrO_2_@NCM85 sample series. The arrows and drop lines indicate the expected Bragg positions (*R*−3 *m* space group). b) X‐ray photoelectron spectroscopy (XPS) data (Zr 3d core‐level region) collected from the uncoated and coated materials. The dashed and dotted lines denote the shift in binding energy between the peak maxima for Z250 and Z700.

To facilitate characterization of the protective coating while maintaining good cyclability, the 15 ALD cycle sample (with ≈1.2 wt% ZrO_2_) was selected for post‐treatment in O_2_ at temperatures of 300, 400, 550, and 700 °C (hereafter referred to as Z300–Z700, with Z250 denoting the as‐coated ZrO_2_@NCM85). The crystal structure of the resulting CAMs was examined by X‐ray diffraction (XRD, **Figure** **2**a). The experimental patterns can be indexed in the space group *R*−3 *m*. Furthermore, Rietveld refinement analysis of the diffraction data indicated that the bulk structure remains virtually unaffected by ALD coating and annealing (see Table S1 and Figure S2,Supporting Information).

The surface of the ZrO_2_@NCM85 CAMs was probed using X‐ray photoelectron spectroscopy (XPS). Figure 2b shows detailed spectra of the Zr 3d core‐level regions. The presence of ZrO_2_ (Zr^4+^) is evident from the characteristic doublet peak, with the 3d_5/2_ line centered at ≈181.8 eV and the 3d_3/2_ line at ≈184.2 eV for Z250, Z300, and Z400. The spectrum barely changed upon annealing at moderate temperatures, suggesting a similar chemical environment of the zirconium ions at the surface. However, there was a distinct peak shift toward lower binding energies for Z550 and Z700, possibly indicating a reaction and/or interaction of the coating with the NCM85 substrate. Recent reports on similar systems have shown that this decrease in binding energy results from the formation of lithium metal oxides.^[^ ^37^ ^]^ Such compounds are believed to be beneficial to the CAM performance, due to superior ionic conductivity over the respective (Li‐free) binary oxides. Nevertheless, it should be noted that Zr^4+^ diffusion into the outermost layer of the NCM85 would also result in a detectable change in the zirconium environment.

**Figure 3:**
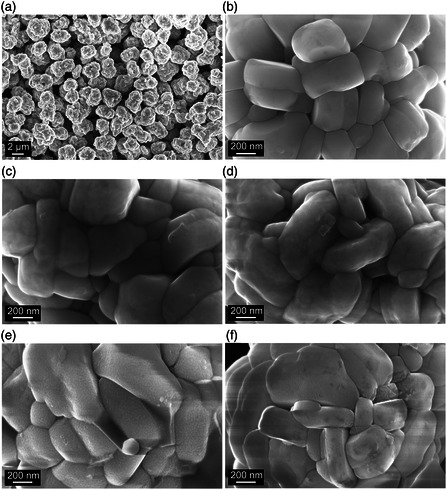
a–f) Top‐view scanning electron microscopy (SEM) images of: the uncoated NCM85 (a,b) and ZrO_2_@NCM85 (c–f) CAMs, with Z250 (c), Z400 (d), Z550 (e), and Z700 (f).

The different samples were further investigated by scanning electron microscopy (SEM, **Figure** **3**a–f). Neither coating nor post‐annealing was found to have a direct effect on the CAM morphology. Closer inspection at higher magnification revealed the presence of a surface shell. In addition, increasing surface roughness was noticed for Z550 and Z700, suggesting an altered coating microstructure and decreasing uniformity above a certain threshold temperature.

**Figure 4:**
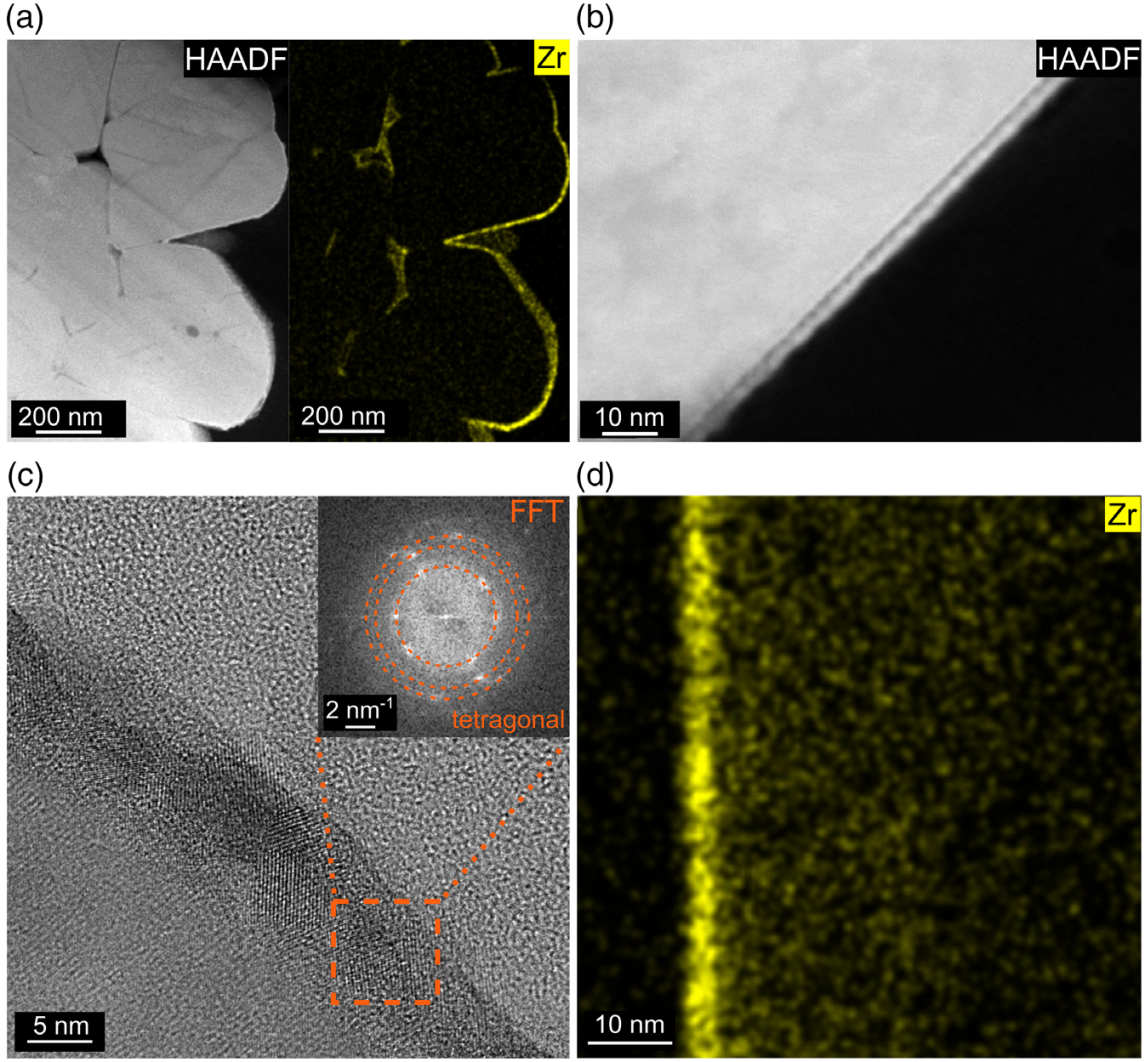
Electron microscopy of focused ion beam (FIB)‐prepared particle cross‐sections of the ZrO_2_@NCM85 CAMs. a) Low‐magnification high‐angle annular dark‐field scanning TEM (HAADF‐STEM) image of Z550 and corresponding elemental map of Zr. b) High‐magnification HAADF‐STEM image showing the surface layer for Z400. c) HRTEM image of Z250 with fast Fourier transform (FFT) pattern (inset) of the crystalline coating. d) Zr elemental map collected from Z700.

Furthermore, transmission electron microscopy (TEM) imaging was done on focused ion beam (FIB)‐prepared particle cross‐sections to gain more detailed insights into the nature of the nanocoating (**Figure** **4**a–d). Uniform secondary particle coverage was confirmed by high‐angle annular dark‐field scanning TEM (HAADF‐STEM) combined with energy‐dispersive X‐ray spectroscopy (EDS). Deposition onto the grain boundaries in the particle interior was not observed. At first glance, the Zr signal in Figure 4a may suggest otherwise, but it originated from areas (on the secondary particle surface) that were not removed during FIB preparation. The coating had a thickness of 4–5 nm, thus exceeding the 2.9 nm estimate, as expected. HAADF‐STEM corroborated the SEM observation of increasing surface roughness with increasing annealing temperature (see Figure 4b and Figure S3, Supporting Information). High‐resolution TEM (HRTEM) investigations indicated that the coating consists of tetragonal ZrO_2_ nanoparticles (Figure 4c) (space group *P*4_2_/*nmc*, see fast Fourier transform (FFT) pattern in the inset), which grow with increasing post‐treatment temperature, from 4 to 5 nm for Z250 to 7 to 8 nm for Z550 (see Figure S4, Supporting Information). This kind of crystal growth/coalescence apparently gives rise to increasing surface roughness. After heating at 550 °C, some of the crystals appeared to have undergone a phase transition toward monoclinic ZrO_2_. This is indicated by an additional reflection in the FFT pattern pointing at the baddeleyite structure (space group *P*2_1_/*c*, see Figure S5, Supporting Information). We note that the phase transformation is in agreement with the dependency of the ZrO_2_ crystal structure on particle size.^[^ 43, 44 ^]^ Surprisingly, the crystallinity of the protective coating was partially lost at 700 °C (see Figure S6, Supporting Information). From the data, it appears that an amorphous layer formed in some regions of the surface that cannot be easily distinguished from the bulk NCM85. Unlike other studies, no evidence for the formation of crystalline Li_2_ZrO_3_ was found.^[^ ^26^ ^]^ Instead, zirconium diffused into the subsurface volume of the layered oxide, as indicated by EDS (Figure 4d).

**Figure 5:**
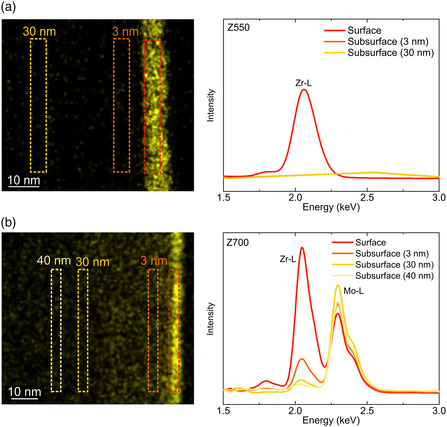
a,b) Zr elemental maps for FIB‐prepared particle cross‐sections of Z550 (a) and Z700 (b) with corresponding spectra from STEM‐EDS in the energy range of the Zr–L edge. The colored rectangles indicate the areas on and below the NCM85 surface, where the spectra were acquired. The Mo signal originates from the sample holder.

The zirconium concentration was found to decrease with increasing depth from the NCM85 (outer) surface (**Figure** **5**a,b), eventually reaching the physical detection limit after ≈40 nm. The diffusion coefficient (700 °C) was estimated at *D* ≈ 3.8 × 10^−16^ cm^2^ s^−1^ from the corresponding STEM‐EDS data (see Supporting Information for details). In contrast, there was no (detectable) zirconium diffusion for annealing temperatures of *ϑ* ≤ 550 °C. Zr^4+^ doping of layered Ni‐rich cathodes at elevated temperatures (starting from NCM with a Zr‐containing surface layer) has been reported previously.^[^ 26, 45 ^]^ In a study by Aurbach et al., diffusion into LiNi_0.8_Co_0.1_Mn_0.1_O_2_ was observed at 700 °C.^[^ ^45^ ^]^ In another work on ALD‐derived ZrO_2_ coatings on LiNi_0.6_Co_0.2_Mn_0.2_O_2_, the authors found signs of minor diffusion into the outer surface of the CAM at 500 °C already.^[^ ^26^ ^]^ While these studies reported on bulk diffusion (on the micrometer scale) with annealing of the coated materials at *ϑ* ≥ 700 °C, the lower diffusion depths observed here probably result from shorter dwell times. Overall, our findings agree with the trend that (Zr‐based) coatings can act as dopants, at least to some degree, depending on the temperature.

**Figure 6:**
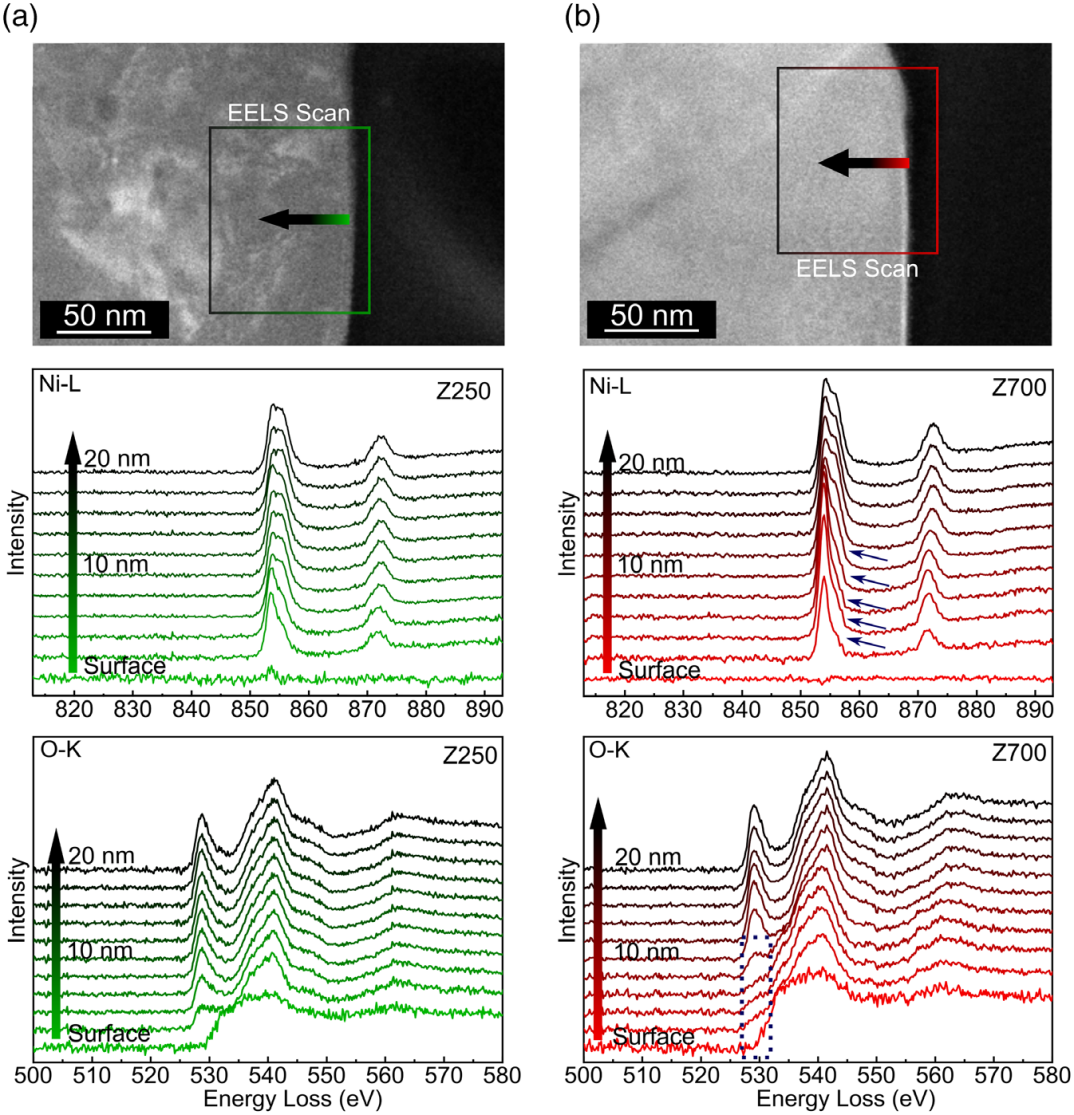
a,b) Electron energy loss (EEL) spectra at the Ni–L and O–K edges of the NCM85 (sub)surface for Z250 (a) and Z700 (b). The green and red squares indicate the areas probed. Spectra were recorded every 2 nm. Evident changes to the near‐surface region of the CAM annealed at 700 °C are highlighted.

The TEM characterization was further complemented by electron energy loss spectroscopy (EELS) studies to learn about potential reactions/interactions between coating and CAM. Specifically, STEM‐EELS measurements were conducted on both Z250 and Z700, focusing on the Ni and O states in the surface and subsurface regions of the NCM85 (**Figure** **6**a,b). The spectra collected from Z700 showed clear signs of altered chemical environments. The pre‐peak of the O‐K edge at ≈529 eV was strongly suppressed over the first 8 nm from the surface toward the bulk (denoted by the dotted rectangle), while this only applied to 2 nm for Z250. The suppression of the pre‐peak, which represents a transition into a σ* hybrid orbital of O 2p and Ni 3d states, was suggested to originate from the presence of oxygen vacancies.^[^ ^46^ ^]^ These vacancies result from oxygen release, which becomes increasingly favorable with progressing delithiation.^[^ ^47^ ^]^ Recently, (sub)surface oxygen loss during battery operation has been identified as a degradation mechanism in Li(Ni_1/3_Co_1/3_Mn_1/3_)O_2_ cathodes using a Li_2_S–P_2_S_5_ SE.^[^ ^48^ ^]^ For Z700, this might be due to maintaining charge balance upon lithium migration into or reaction with the ZrO_2_, which has been reported previously for protective alumina coatings on NCM.^[^ ^37^ ^]^ The spectra also showed partial suppression of the right shoulder of the Ni–L3 edge (≈855.5 eV, denoted by blue arrows) and a more pronounced low‐loss portion of the peak (≈854 eV) compared with Z250, especially in the first 10 nm. This is indicative of surface Ni reduction and formation of a rocksalt‐like NiO layer, which could be explained by the aforementioned oxygen release.^[^ ^49^ ^]^ The presence of such an insulating phase has been shown to negatively affect the cycling performance.^[^ ^47^ ^]^ Apart from that, a structurally distorted (dislocations, etc.) interlayer between coating and CAM was found by high‐resolution STEM (see Figure S7, Supporting Information).

**Figure 7:**
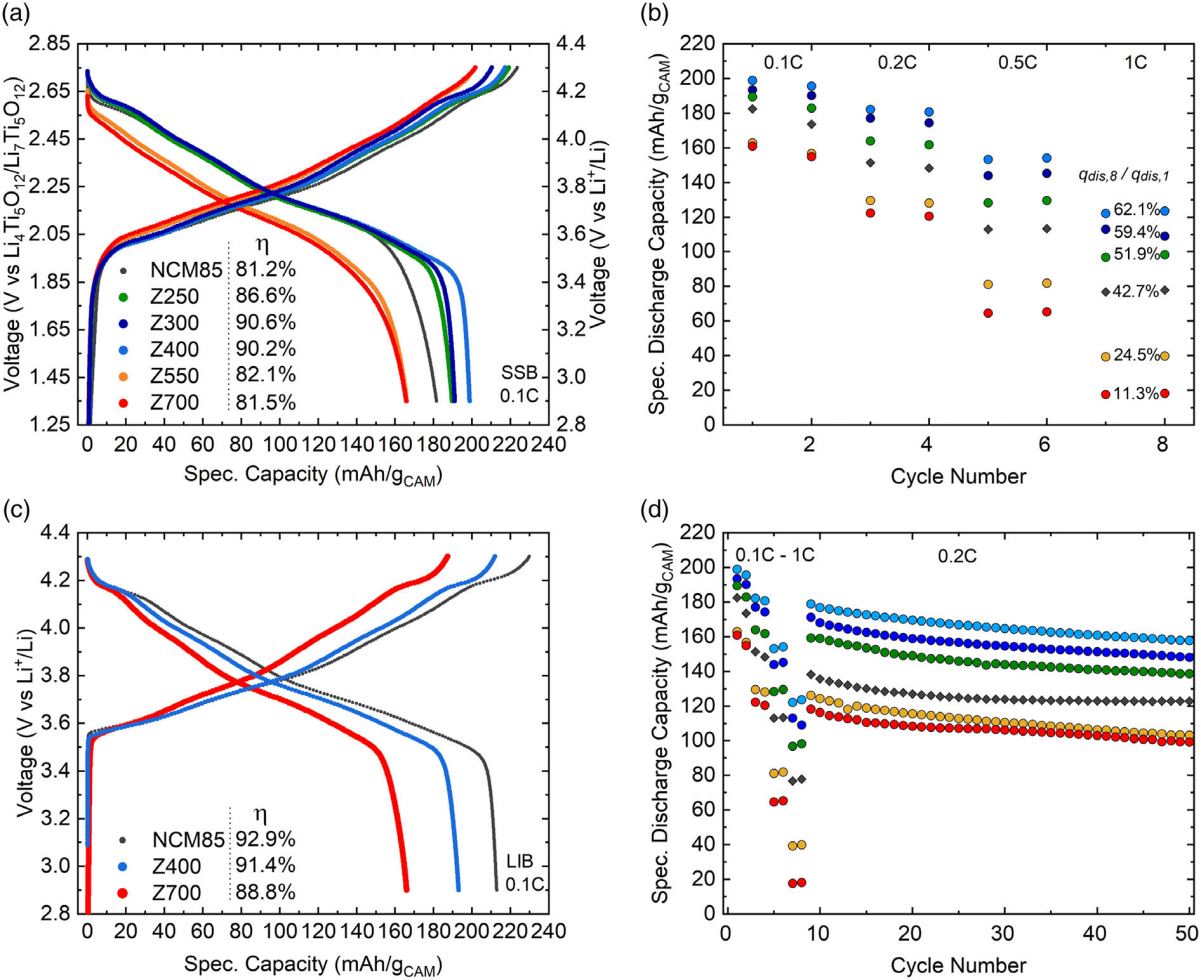
Electrochemical performance of the uncoated NCM85 and ZrO_2_@NCM85 CAMs in solid‐state battery (SSB) cells at 45 °C in the voltage range 1.35–2.75 versus Li_4_Ti_5_O_12_/Li_7_Ti_5_O_12_ (≈2.9–4.3 V vs Li^+^/Li). a) First‐cycle voltage profiles at 0.1C and corresponding Coulomb efficiencies. b) Specific discharge capacities during rate testing and capacity ratios for the 8th cycle@1C/1st cycle@0.1C. c) First‐cycle voltage profiles for the uncoated NCM85, Z400, and Z700 in LIB cells at 0.1C and 45 °C in the voltage range 2.9–4.3 V versus Li^+^/Li and corresponding Coulomb efficiencies. d) Specific discharge capacities during rate testing (see (b)) and subsequent cycling at 0.2C.

The materials prior to and after coating were tested electrochemically in bulk‐type SSBs at 45 °C using argyrodite LPSCl (*σ*_ion_ ≈ 2 mS cm^−1^ at room temperature) as SE in the separator and the electrodes. LTO served as anode‐active material in this work. XRD patterns of both LPSCl and LTO are shown in Figure S8, Supporting Information. The cells were cycled in the voltage range 1.35–2.75 V (≈2.9–4.3 V vs Li^+^/Li) to explore the influence of the CAM coating and post‐treatment on the cycling performance and stability. **Figure** **7**a shows the voltage profiles of representative cells in the initial cycle at 0.1C. As expected, the specific charge capacity was reduced for the ZrO_2_@NCM85 samples, especially for Z550 and Z700. However, the reversible capacities achieved with Z250, Z300, and Z400 were larger than that of the uncoated CAM, with up to 199 mAh g_CAM_^−1^ for Z400, corresponding to ≈2.1 mAh cm^−2^. The reason is the higher Coulomb efficiency of cells using these materials (81.2% for uncoated NCM85, compared to 86.6% for Z250 and >90% for Z300 and Z400) or, in other words, the lower tendency for SE degradation (oxidation), a well‐documented problem of thiophosphate‐based SSBs.^[^ 6, 7, 8, 9, 50 ^]^ The increase in Coulomb efficiency with annealing at 250 °C < *ϑ* ≤ 400 °C suggests more effective surface passivation, probably because of densification of the coating, and points at a high degree of coverage of the NCM85 surface.^[^ 7, 8 ^]^ While low electronic conductivity is a desirable property of coatings for mitigating interfacial degradation, loss of electronic contact between CAM particles must be avoided.^[^ ^7^ ^]^ Interestingly, the electronic conductivity in the composite cathode was not affected much compared with the uncoated NCM85, despite the relatively dense nature of the ALD‐derived ZrO_2_ nanocoating. The voltage profiles further revealed that the “additional” discharge capacity for Z250, Z300, and Z400 can be attributed to improved lithiation at low voltages. This is also apparent from the peak at ≈1.95 V in the corresponding differential capacity curves (see Figure S9, Supporting Information). The presence of this peak depends upon the C‐rate; hence, it is clearly associated with the kinetics of the re‐intercalation reaction. Cells using Z550 and Z700 delivered considerably lower specific capacities due to high overpotentials, as well as irreversibilities similar to the uncoated NCM85.

The cycling performance of the ZrO_2_@NCM85 CAMs was tested at rates ranging from 0.1C to 1C, corresponding to current densities of ≈0.2 and ≈2 mA cm^−2^, respectively. The general trends (Figure 7b) were similar to that observed in the initial cycle. While the charge‐storage properties improved with coating and post‐annealing at moderate temperatures, they were adversely affected for both Z550 and Z700. The differences in cell capacity increased with increasing current density. At 1C, Z400 performed best in absolute and relative terms, with a specific discharge capacity of 124 mAh g_CAM_^−1^ (≈62% of the initial capacity at 0.1C), compared to 78 mAh g_CAM_^−1^ (≈43%) for uncoated NCM85 and only 18 mAh g_CAM_^−1^ (≈11%) for Z700. The differences in rate capability can be explained in part by the level of side reactions. This holds for the improvements observed for Z300 and Z400 over Z250. Oxidative SE degradation has been shown to increase the cathode resistance, which is directly reflected in the capacity, especially at high rates.^[^ 7, 50 ^]^ However, the poor performance of both Z550 and Z700 cannot be explained by unwanted side reactions only. Likewise, their behavior cannot be explained by the presence of abundant unprotected CAM, as argued in previous studies.^[^ ^26^ ^]^ The latter would not be expected to result in a worse performance than for the uncoated NCM85. Furthermore, Zr^4+^ doping, which occurs above a certain threshold temperature, has been reported to be beneficial to the cyclability of layered metal oxides and other CAMs.^[^ 45, 51 ^]^ Instead, the performance decay appears to be due to impeded ion transport. These considerations led to the STEM‐EELS investigations described above, revealing the formation of a rocksalt‐like NiO layer and helping to explain the resistive nature of the modified CAM surface. The CAM‐inherent performance limitations are corroborated by their cyclability in liquid electrolyte‐based LIB cells (Figure 7c). Z700 was found to deliver a similarly low first‐cycle specific capacity under identical conditions. Notably, the phase transitions occurred at the same voltages for the uncoated NCM85, Z400, and Z700. This result thus suggests that redox inactivity rather than overpotential (as in the case of SSB cells) plays a role in the differences seen in capacity among the samples. Apart from that, a comparison of the Coulomb efficiencies confirmed that SSB cells using the uncoated NCM85 suffer strongly from (electro)chemical side reactions at the SE|CAM interface in the initial cycle.

After rate performance testing, the SSB cells were subjected to cycling at 0.2C. As can be seen from the data in Figure 7d, only Z250, Z300, and Z400 were capable of delivering larger specific capacities than the uncoated CAM (by 16–35 mAh g_CAM_^−1^ in the 50th cycle), which stabilized at 124 mAh g_CAM_^−1^ after 30 cycles. They also showed a more gradual fading with cycling. Taken together, the results demonstrate that good cycling performance is achievable by proper post‐treatment, without adding an additional lithium source during coating or the presence of abundant Li_*x*_Zr_*y*_O_*z*_.

**Figure 8:**
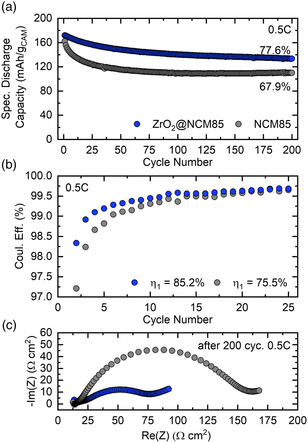
Long‐term cycling performance of the uncoated NCM85 and a selected ZrO_2_@NCM85 CAM (10 ALD cycle sample, 350 °C post‐annealing) in SSB cells at 0.5C and 45 °C in the voltage range 1.35–2.75 versus Li_4_Ti_5_O_12_/Li_7_Ti_5_O_12_ (≈2.9–4.3 V vs Li^+^/Li). a) Specific discharge capacities over 200 cycles. b) Coulomb efficiencies for cycles 2–25. The first‐cycle values are denoted in the figure. c) Nyquist plots of the electrochemical impedance of representative cells after 200 cycles.

The long‐term cycling performance of SSB cells using a selected ZrO_2_@NCM85 CAM (10 ALD cycle sample, 350 °C post‐annealing) was studied at a rate of 0.5C in some more detail. This sample was chosen based on initial results from electrochemical testing. Nevertheless, its cyclability is very similar to that of Z300/Z400. As expected from the aforementioned data, the coated material delivered a larger initial specific discharge capacity (172 vs 164 mAh g_CAM_^−1^) and showed better capacity retention (≈78% vs ≈68% of the initial capacity after 200 cycles) than the uncoated NCM85 (**Figure** **8**a). Similar to the cycling at 0.2C, the fading was more gradual compared to that of the uncoated CAM, where the majority of the capacity degradation occurred over the first 25 cycles, due to increasing overpotential resulting from side reactions at the SE|CAM interface. The latter is corroborated by the data shown in Figure 8b. The Coulomb efficiency of cells with the ZrO_2_@NCM85 CAM was much higher during the first few cycles (e.g., 85.2% vs 75.5% in the initial cycle). The different extent of SE oxidation was also evident from electrochemical impedance spectroscopy (EIS) analysis. Semi‐quantitative comparison of Nyquist plots of the electrochemical impedance for the respective cells after 200 cycles (Figure 8c) revealed a difference in cathode interfacial resistance by a factor of about two (EIS data collected prior to cycling are shown in Figure S10, Supporting Information).

**Figure 9:**
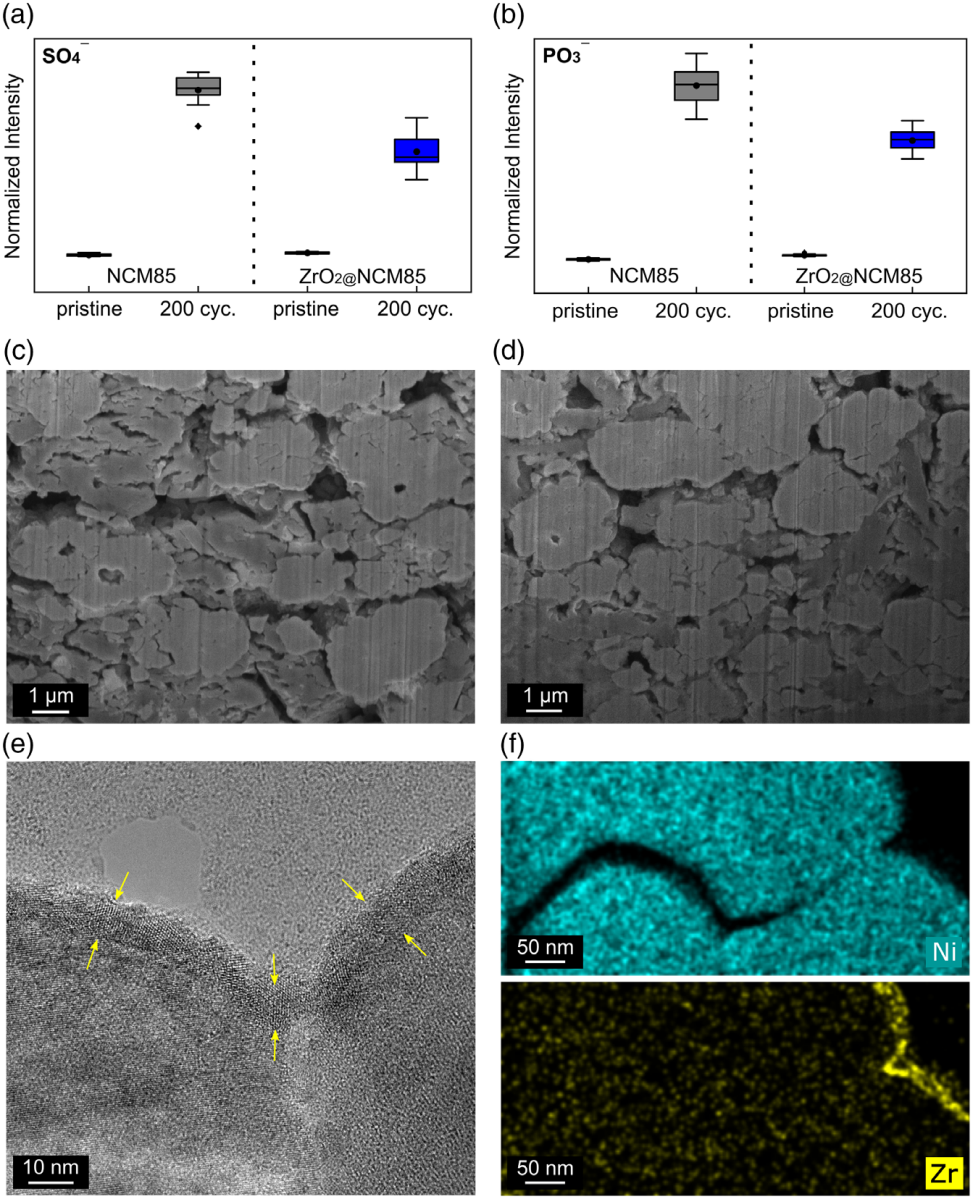
Chemical and morphological analysis of the SSB cathodes after 200 cycles at 0.5C and 45 °C. a,b) Box plots of measured intensities for PO_3_^−^ and SO_4_^−^ fragments from time‐of‐flight secondary‐ion mass spectrometry (ToF‐SIMS). c,d) Cross‐sectional SEM images of electrodes containing: c) the uncoated NCM85 and d) ZrO_2_@NCM85 (10 ALD cycle sample, 350 °C post‐annealing). e) HRTEM image with arrows indicating the intact coating. f) Elemental maps of Ni and Zr collected from a FIB‐prepared cross‐section of a ZrO_2_@NCM85 particle.

Time‐of‐flight secondary‐ion mass spectrometry (ToF‐SIMS) was applied to link the electrochemical data with the interfacial degradation. Investigations on electrodes prior to and after cycling (200 cycles at 0.5C and 45 °C) provided direct evidence of a larger amount of oxygen‐containing phosphorus and sulfur species in cycled SSB cathodes containing the uncoated NCM85. The measured intensities for the PO_3_^−^ and SO_4_^−^ fragments are shown in **Figure** **9**a,b (see Figure S11, Supporting Information for other species). Such species are formed exclusively at the SE|CAM interface, since the NCM85 represents the only oxygen source in the system. While mitigating interfacial degradation, complete suppression could not be achieved by ALD coating of the CAM secondary particles. This result agrees well with the large number of available literature reports on (oxide) coatings, from which no conclusion can be drawn as to what composition/morphology is most effective in suppressing side reactions.^[^ 6, 7, 12 ^]^ Interestingly, there were no significant differences in the amount of polysulfide species, which can probably be explained by the fact that they are also formed at the interfaces of the SE with the current collector and carbon black.

In addition to the chemical degradation, the mechanical integrity of the CAMs was studied by cross‐sectional SEM of FIB‐prepared SSB cathodes prior to (see Figure S12, Supporting Information) and after cycling (see Figure 9c,d and Figure S13, Supporting Information). After 200 cycles, both the coated and uncoated samples showed signs of particle fracture typical of polycrystalline Ni‐rich NCM due to anisotropic volume variations upon (de)lithiation. The cracking occurred primarily at the grain boundaries of the primary particles. Visual/qualitative comparison indicated similar or slightly lower mechanical degradation for the ZrO_2_@NCM85, thereby suggesting that the coating helps indirectly to provide some degree of stabilization. Note that Han et al. showed that particle fracture in SSB cells is also determined by the (electro)chemical side reactions occurring at the SE|CAM interface.^[^ ^52^ ^]^ For more detailed insights into the mechanical behavior, other techniques, such as tomography and/or specific electrochemical methods, would be required. In general, it is difficult to suppress the cracking of polycrystalline NCM CAMs,^[^ ^7^ ^]^ which explains the increasing interest in their single‐crystalline counterparts.^[^ 53, 54, 55, 56 ^]^

Although the evolution of the SE|CAM interface is known to have a considerable effect on battery performance, it is challenging to visualize, requiring inert and cryo conditions during preparation and imaging. Apart from that, in situ/operando investigations would be desirable to rule out relaxation effects.^[^ ^57^ ^]^ However, mechanical degradation probably played a minor role in the capacity decay considering the relatively low state‐of‐charge achieved under the cycling conditions used here. The coating (morphology, crystallinity, and conformity) was preserved well after cycling, as is evident from the HRTEM image and elemental maps in Figure 9e,f. This is notable because coating segregation, aggregation, and ”breakdown” (e.g., for Li_2_S–P_2_S_5_|Li_2_ZrO_3_@LiNi_0.5_Co_0.2_Mn_0.3_O_2_)^[^ ^21^ ^]^ as well as exfoliation (e.g., for Li_3_PS_4_|LiNbO_3_@LiNi_1/3_Co_1/3_Mn_1/3_O_2_)^[^ ^48^ ^]^ have been identified as degradation modes in the past. Stable contact between CAM and coating is crucial for maintaining good battery performance, given that separation would create a physical barrier for charge transport and could lead to local oxygen release, as shown recently.^[^ ^48^ ^]^ Furthermore, delithiation of Li_2_ZrO_3_ at voltages ≥4.3 V versus Li^+^/Li has been demonstrated,^[^ ^21^ ^]^ making more inert binary oxide coatings, such as ZrO_2_, advantageous over Li‐containing (ternary) ones.

## 3 Conclusion

ZrO_2_‐modified NCM85 CAMs were characterized for use in bulk‐type SSB cells. Conformal secondary particle coatings were grown by ALD. Such prototype (protective) nanocoatings allow investigating how their properties affect the cycling performance without the confounding influence of other factors, such as ill‐defined morphology or nonuniform coverage. Investigations into the effect of post‐heat treatment revealed: 1) nanocrystal growth below 550 °C; 2) partial transformation from tetragonal to monoclinic ZrO_2_ at 550 °C and amorphization at higher temperatures; 3) ion diffusion into subsurface regions of the NCM85 at 700 °C; as well as 4) formation of a rocksalt‐like NiO layer (along with a distorted interlayer) above 550 °C, due to unfavorable interactions between coating and CAM. The modified NCM85 after “mild” annealing exhibited a promising cycling performance. High specific discharge capacities of 199 and 124 mAh g_CAM_^−1^ at ≈0.2 and ≈2 mA cm^−2^, respectively, and a good cycling stability with 77.6% capacity retention after 200 cycles at ≈1 mA cm^−2^ were achieved. Unlike reported previously, the improvement in cyclability did not result from the formation of a ternary oxide coating, but instead from more effective surface passivation. Post‐mortem analysis revealed that the extent of SE oxidation is lower for the modified NCM85, while there were only minor differences in mechanical integrity between the coated and uncoated CAM particles. The coating morphology and crystallinity were found to be retained after cycling.

Overall, this study provides insights into the processes occurring during the preparation/post‐modification of Zr‐based electrode coatings and their effect on the battery performance. It highlights the overlapping phenomena that need to be considered when applying a secondary particle coating for stabilizing cathode interfaces in thiophosphate‐based SSB cells. To the best of our knowledge, there is still no protective coating available that allows operation with high Coulomb efficiency beyond 99.9%, low overpotential, and low capacity fading, all of which is needed to compete with the cathode performance in state‐of‐the‐art LIBs.

## 4 Experimental Section

4.1

4.1.1

##### Cathode Coating and Post‐Treatment

Prior to the coating process, LiNi_0.85_Co_0.10_Mn_0.05_O_2_ (NCM85, *d*_50_ ≈ 3.5 μm, *d*_90_ ≈ 5.0 μm; BASF SE) CAM was heated for 3 h in O_2_ flow at 750 °C (heating and cooling rates set to 5 °C min^−1^). For the ZrO_2_ deposition, 2–8 g NCM85 CAM was encased in a gas‐permeable powder holder and introduced into the ALD reactor (Picosun), followed by flushing with N_2_ and stabilization at 250 °C for 1 h. The coating process comprised 2–15 ALD cycles, with the same pulse sequence for O_3_ (ozone generator from IN USA Inc.) and Zr[N(CH_2_CH_3_)(CH_3_)]_4_ (TEMAZ, 99.99%; Sigma‐Aldrich) and the precursor container heated at 105 °C. A typical sequence consisted of 100 pulses of 0.1 s duration, each followed by 2 s reactor purging and 60 s purging after the last pulse. The N_2_ carrier‐gas flow in the TEMAZ and ozone lines was set to 200 sccm. Finally, the ZrO_2_@NCM85 CAM was heated in O_2_ flow at temperatures ranging from 300 to 700 °C for 30 min (heating and cooling rates set to 10 °C min^−1^).

##### Elemental Analysis

For compositional analysis, the ZrO_2_@NCM85 CAM was dissolved in acid using a pressurized and heated digestion system. The Zr content was determined by ICP‐OES using a Thermo Fisher Scientific iCAP 7600 DUO. The carbon content was probed using a CS analyzer.

##### XRD

XRD data were collected in Debye–Scherrer geometry using an STOE Stadi‐P diffractometer with a DECTRIS MYTHEN 1K strip detector and a Mo anode (*λ* = 0.70926 Å). The instrumental contribution to the peak broadening was determined by measuring a NIST 660c LaB_6_ standard as a line broadening reference. Rietveld refinement analysis was performed using the software FullProf. NCM structural models were refined against the data, where the scale factor, zero shift, and peak shape parameters *U* and *Y* were allowed to vary. In the structural model, the unit‐cell parameters, oxygen *z*‐coordinate, isotropic temperature factors (*B*_iso_) of the oxygen and transition‐metal atoms (with fixed *B*_iso,Li_ = 1.0 Å^2^), and occupancy of the Li site were refined. All sites were constrained to be fully occupied and atoms occupying the same site were constrained to have the same atomic parameters. The confidence intervals were determined by multiplying error outputs from the program by a factor of three.

##### XPS

XPS measurements were carried out on a PHI VersaProbe II instrument (ULVAC‐PHI, Inc.) using monochromatic Al‐Kα radiation (*E* = 1486.6 eV). The power of the X‐ray source was set to 100 W. The powder samples were pressed into a Teflon cup holder, which was mounted on a sample holder using insulating double‐sided tape (inert sample transfer). The examined area had a size of 1.3 mm × 0.1 mm. For survey and detailed spectra, pass energies of 117.4 and 23.5 eV, respectively, were used. For charge neutralization, the PHI dual‐beam charge neutralization was employed, consisting of a 20 nA and 10 V Ar‐ion beam in combination with a 20 μA and 3 V electron beam and effectively pinning the sample potential at −3 V versus ground potential. Data evaluation was done with the software CasaXPS (version 2.3.25; Casa Software Ltd.). The spectra were calibrated in relation to the signal of adventitious carbon at 284.8 eV. A Shirley background was used and the spectra were fitted assuming GL(30) line shape.

##### SEM

SEM analysis was carried out using an LEO‐1530 electron microscope (Carl Zeiss AG) with a field emission source at an accelerating voltage of 10 kV. Cross‐sections of pristine cathodes were prepared by ion milling using an IB‐19510CP cross‐section polisher (JEOL). Cross‐sections of cycled cathodes were prepared and examined using a Strata 400S (FEI Company).

##### TEM

TEM specimens were prepared by the lift‐out technique using a Strata 400S focused Ga‐ion beam. The area of interest was coated with a carbon film to protect the samples from beam damage. The samples were milled and thinned at 30 kV and polished at 5 and 2 kV to clean the surface. The prepared lamellae were attached to Mo or Cu half grids for the TEM characterization. A probe‐corrected Themis 300 TEM and a double aberration‐corrected Themis Z TEM (Thermo Fisher Scientific) were used to image the specimens, both at an accelerating voltage of 300 kV. HAADF‐STEM images were collected using a HAADF detector with a collection semi‐angle range from 60 to 200 mrad. The EDS mapping was done using an EDAX SuperX EDS detector. EELS data were acquired by a Gatan image filter with a K3 camera (Gatan Inc.) using a camera length of 29.5 mm, a screen current of 20 pA, and an energy dispersion of 0.18 eV/channel. The energy resolution estimated from the FWHM of the zero‐loss peak is ≈1.7 eV. The NBED patterns were acquired in micro‐STEM mode with a convergence semi‐angle of 0.2 mrad and a camera length of 380 mm.

##### ToF‐SIMS

ToF‐SIMS measurements were performed on a TOF‐SIMS 5–100 system (IONTOF GmbH). The samples were prepared and transferred to the device under an inert atmosphere. All measurements were performed in negative ion mode using Bi_3_^+^ (25 keV) as primary ions. The surface of the samples was flooded with low‐energy electrons for charge compensation. The cycle time was set to 60 μs. Surface analysis was performed in spectrometry mode (bunched mode) to enable high signal intensities and high mass resolution. The analysis area was 150 μm × 150 μm and rasterized with 256 pixels × 256 pixels (random mode). Every patch was analyzed with 1 frame and 1 shot per pixel and frame. The measurement was stopped after a primary ion dose of 10^12^ ions cm^−2^ was reached (static condition). The primary ion current was ≈0.5 pA. Data evaluation was carried out using the software SurfaceLab 7.1 from IONTOF GmbH.

##### Preparation of Electrode Composites

The cathode composites were prepared by mixing the uncoated NCM85 or ZrO_2_@NCM85 CAMs with LPSCl SE (NEI Corp.), and Super C65 carbon black (Timcal) in a ratio of 69:30:1 by weight using 10 zirconia balls in a planetary mill (Fritsch) at 140 rpm for 30 min under an Ar atmosphere. Analogously, the anode composites were prepared from carbon‐coated LTO (NEI Corp.), LPSCl SE, and Super C65 carbon black at a weight ratio of 30:65:5.

##### SSB Assembly and Testing

The electrochemical performance of the uncoated NCM85 and ZrO_2_@NCM85 CAMs was tested in bulk‐type SSB cells as described previously.^[^ ^39^ ^]^ The separator layer of diameter 10 mm was produced by compacting 100 mg LPSCl SE at a uniaxial pressure of 62 MPa. The pellet stack was completed by adding 65 mg anode composite and 11–12 mg cathode composite (≈2.9 mAh cm^−2^ for *q*_th_ = 274 mAh g_CAM_^−1^) on either side and pressing the stack at 437 MPa. The cells were galvanostatically cycled at 45 °C in a voltage range between 1.35 and 2.75 versus Li_4_Ti_5_O_12_/Li_7_Ti_5_O_12_ while maintaining a uniaxial pressure of 81 MPa. Rate performance tests were carried out at 0.1, 0.2, 0.5, and 1C (with 1C = 190 mA g_CAM_^−1^), with two charge/discharge cycles at each C‐rate, followed by cycling at 0.2C. Stability tests were carried out at 0.5C for 200 cycles.

##### LIB Assembly and Testing

Cathodes for testing in LIB half‐cells were produced by casting an *N*‐methyl‐2‐pyrrolidone (NMP)‐based slurry onto Al foil, followed by vacuum‐drying at 120 °C and calendering at 15 N mm^−1^. The cathodes consisted of CAM, Super C65 carbon black, and polyvinylidene fluoride (PVDF) binder in a 94:3:3 weight ratio. Circular electrodes of 12 mm diameter with an areal loading of ≈8 mg_CAM_ cm^−2^ were punched out from the as‐prepared tapes and used to assemble 2032 coin cells. The cells comprised a glass fiber separator (GF/D; Whatman), 95 μL electrolyte (1m LiPF_6_ in 3:7 by weight ethylene carbonate and ethyl methyl carbonate; BASF SE), and a Li‐metal anode (Albemarle Germany GmbH). They were cycled at 0.1C and 45 °C in the same voltage range as the SSB cells.

##### EIS

EIS was conducted on SSB cells in the discharged state prior to cycling and after 200 cycles at frequencies ranging from 7 MHz to 100 mHz with an amplitude of 10 mV using an SP‐300 impedance analyzer (Bio‐Logic Science Instruments Ltd.)

##### Statistical Analysis

Electrochemical cycling data are averaged from at least three independent measurements. Mean values were determined via “Statistics on Rows” using OriginPro 2018b (OriginLab Corp.). Zr mass fraction data for 5 and 10 ALD cycles are the average from two or more independent experiments on different batches of NCM85 (using the same synthesis parameters). Both mean values and standard deviations were determined via “Statistics on Rows” using OriginPro 2018b (OriginLab Corp.). Mass fractions from ICP‐OES analysis represent the average of three independent measurements on samples from the same batch. For ToF‐SIMS, 13 mass spectra were collected in different areas on the sample surface to ensure the reproducibility of results.

## Conflict of Interest

The authors declare no conflict of interest.

## Supporting information

Supplementary Material

## Data Availability

The data that support the findings of this study are available from the corresponding author upon reasonable request.
