# Supplementary material for: Atomic Layer Deposition Derived Zirconia Coatings on Ni‐Rich Cathodes in Solid‐State Batteries: Correlation Between Surface Constitution and Cycling Performance
Source: Small Sci. 2022 Dec 11;3(2):2200073. doi: 10.1002/smsc.202200073 (PMC11935935; doi:10.1002/smsc.202200073)
Supplement: Supplementary file 1 — Supplementary Material [file SMSC-3-2200073-s001.pdf]

Supporting Information

Atomic Layer Deposition Derived Zirconia Coatings on Ni-Rich Cathodes in Solid-State Batteries: Correlation Between Surface Constitution and Cycling Performance

David Kitsche,[a] Yushu Tang,[b] Hendrik Hemmelmann,[c] Felix Walther,[c] Matteo Bianchini,[a,d] Aleksandr Kondrakov,[a,d] Jürgen Janek[a,c] and Torsten Brezesinski*[a]

[a] D. Kitsche, Prof. Matteo Bianchini, Dr. A. Kondrakov, Prof. J. Janek, Dr. T. Brezesinski

Battery and Electrochemistry Laboratory (BELLA), Institute of Nanotechnology, Karlsruhe Institute of Technology (KIT), Hermann-von-Helmholtz-Platz 1, 76344 Eggenstein-Leopoldshafen, Germany

Email: torsten.brezesinski@kit.edu

[b] Dr. Y. Tang

Institute of Nanotechnology, Karlsruhe Institute of Technology (KIT), Hermann-von-Helmholtz-Platz 1, 76344 Eggenstein-Leopoldshafen, Germany

[c] H. Hemmelmann, Dr. F. Walther, Prof. J. Janek

Institute of Physical Chemistry & Center for Materials Research (ZfM/LaMa), Justus-Liebig-University Giessen, Heinrich-Buff-Ring 17, 35392 Giessen, Germany

[d] Prof. Matteo Bianchini, Dr. A. Kondrakov

BASF SE, Carl-Bosch-Str. 38, 67056 Ludwigshafen, Germany

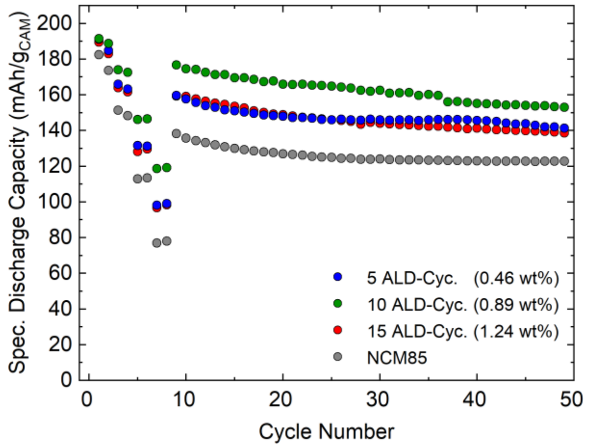

Figure S1. Electrochemical performance of the uncoated NCM85 and (as-coated) ZrO_2@NCM85 CAMs in SSB cells depending on the number of TEMAZ/O_3-ALD cycles. The cells were cycled at 45 °C in the voltage range 1.35-2.75 vs $Li_4Ti_5O_{12}/Li_7Ti_5O_{12}$ (approx. 2.9-4.3 V vs Li^+/Li) at rates ranging from 0.1C to 1C (two cycles each), followed by cycling at 0.2C.

Table S1. Crystallographic parameters from Rietveld refinement of XRD data collected from the uncoated NCM85 and ZrO_2@NCM85 CAMs.

Sample	Rf (%)	*a* (Å)	*c* (Å)	*V* (Å^3)	*z* of O	B_{iso} O site (Å^2)	B_{iso} TM site (Å^2)	Ni^{2+} on Li site (%)
NCM	4.98	2.8671(1)	14.1725(9)	100.892(9)	0.257(1)	0.5(3)	0.11(8)	2.5(0.9)
Z250	5.11	2.8675(1)	14.1734(9)	100.925(9)	0.257(1)	0.6(3)	0.22(9)	2.8(0.9)
Z300	4.48	2.8673(1)	14.1717(9)	100.900(9)	0.257(1)	0.5(3)	0.16(9)	3.2(1.0)
Z400	4.09	2.8675(1)	14.1734(9)	100.929(9)	0.257(1)	0.6(3)	0.17(8)	2.8(0.9)
Z550	4.62	2.8676(1)	14.1752(9)	100.950(9)	0.257(1)	0.5(3)	0.16(9)	2.5(1.0)
Z700	3.81	2.8676(1)	14.1755(9)	100.949(9)	0.257(1)	0.6(3)	0.21(8)	2.8(0.9)

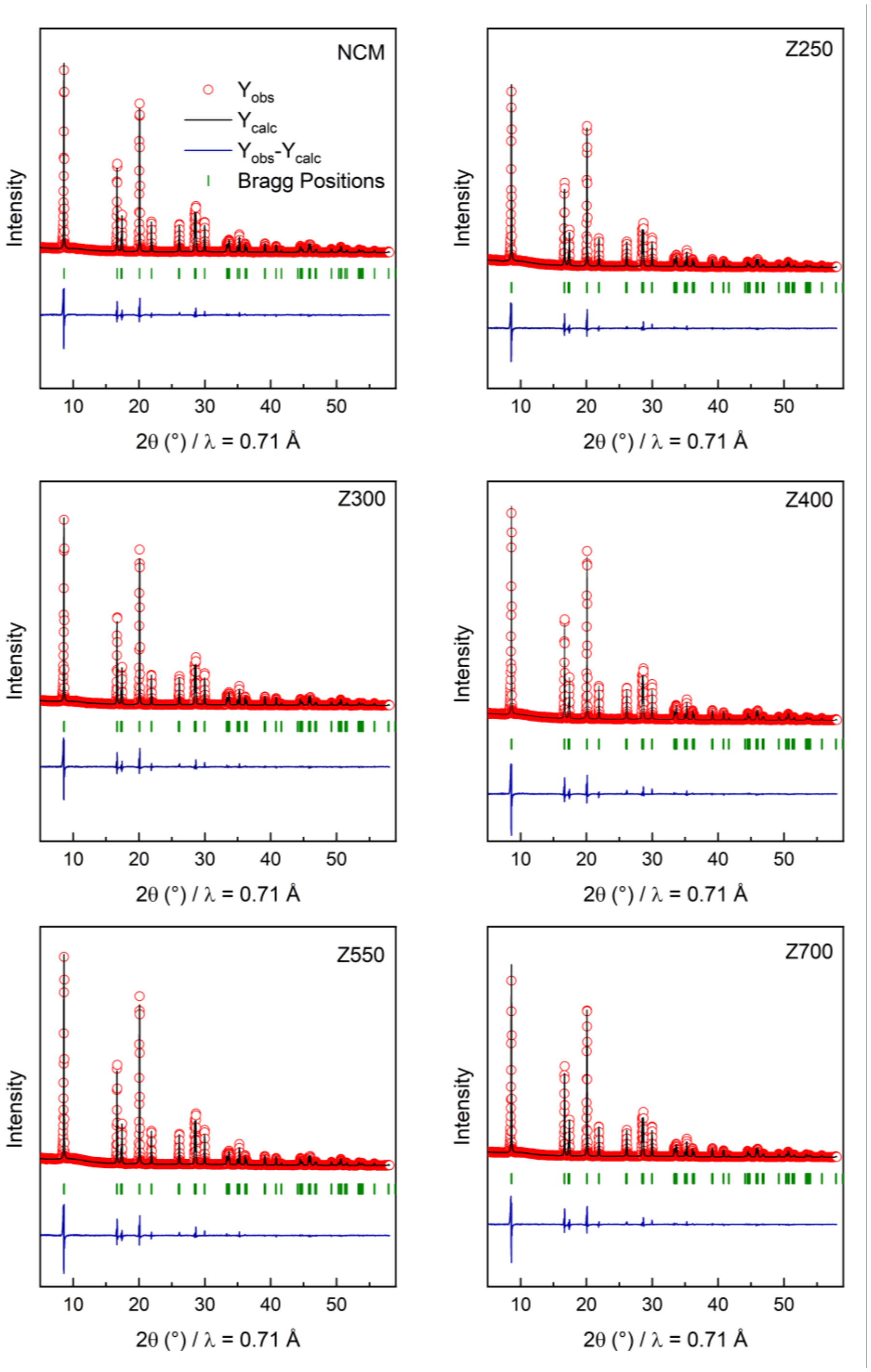

Figure S2. XRD patterns of the uncoated NCM85 and ZrO_2@NCM85 CAMs annealed at different temperatures and corresponding Rietveld refinement profiles.

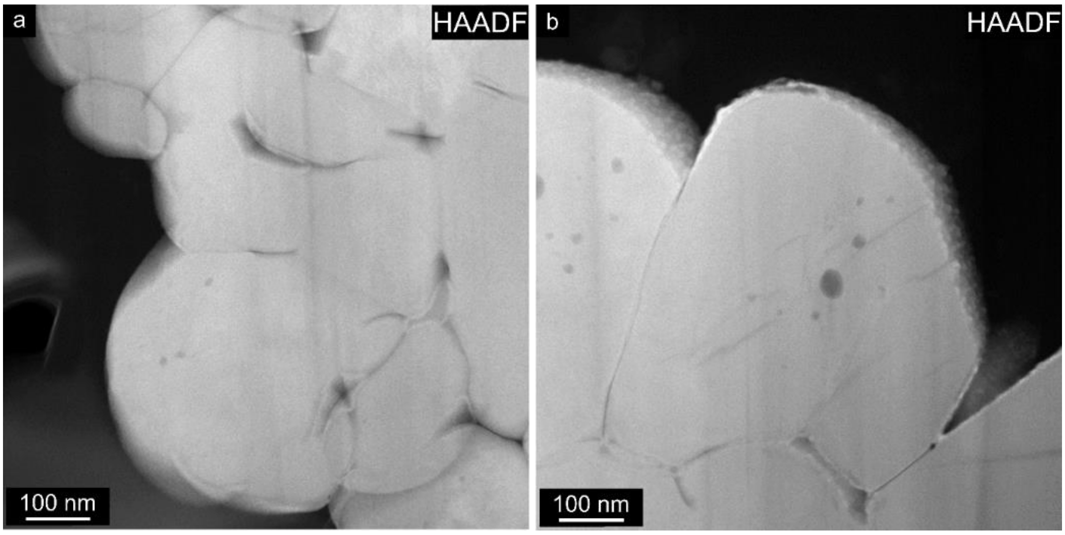

Figure S3. Low-magnification STEM-HAADF images of FIB-prepared particle cross-sections of (a) Z250 and (b) Z550 showing increasing surface roughness.

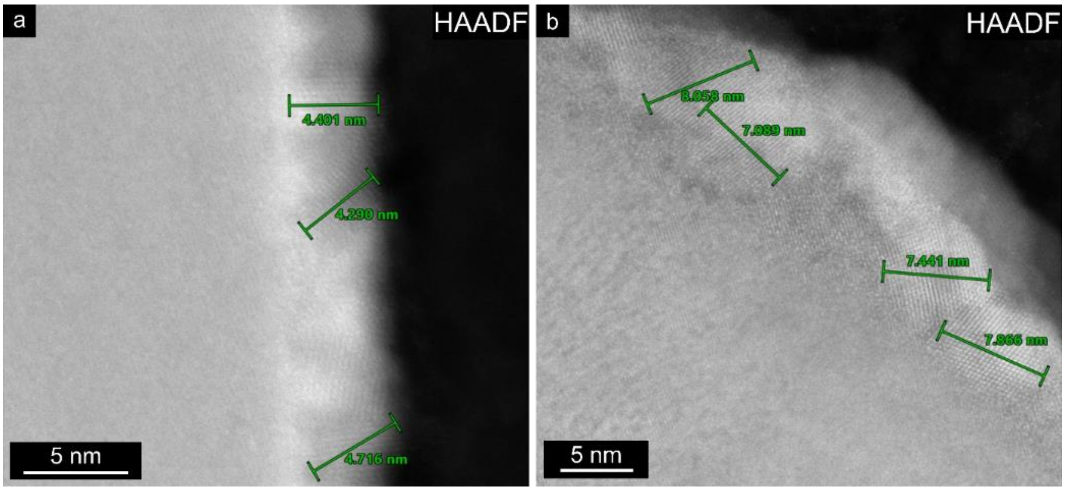

Figure S4. STEM-HAADF images of FIB-prepared particle cross-sections of (a) Z250 and (b) Z550, with indication of the approximate crystal size (ZrO_2).

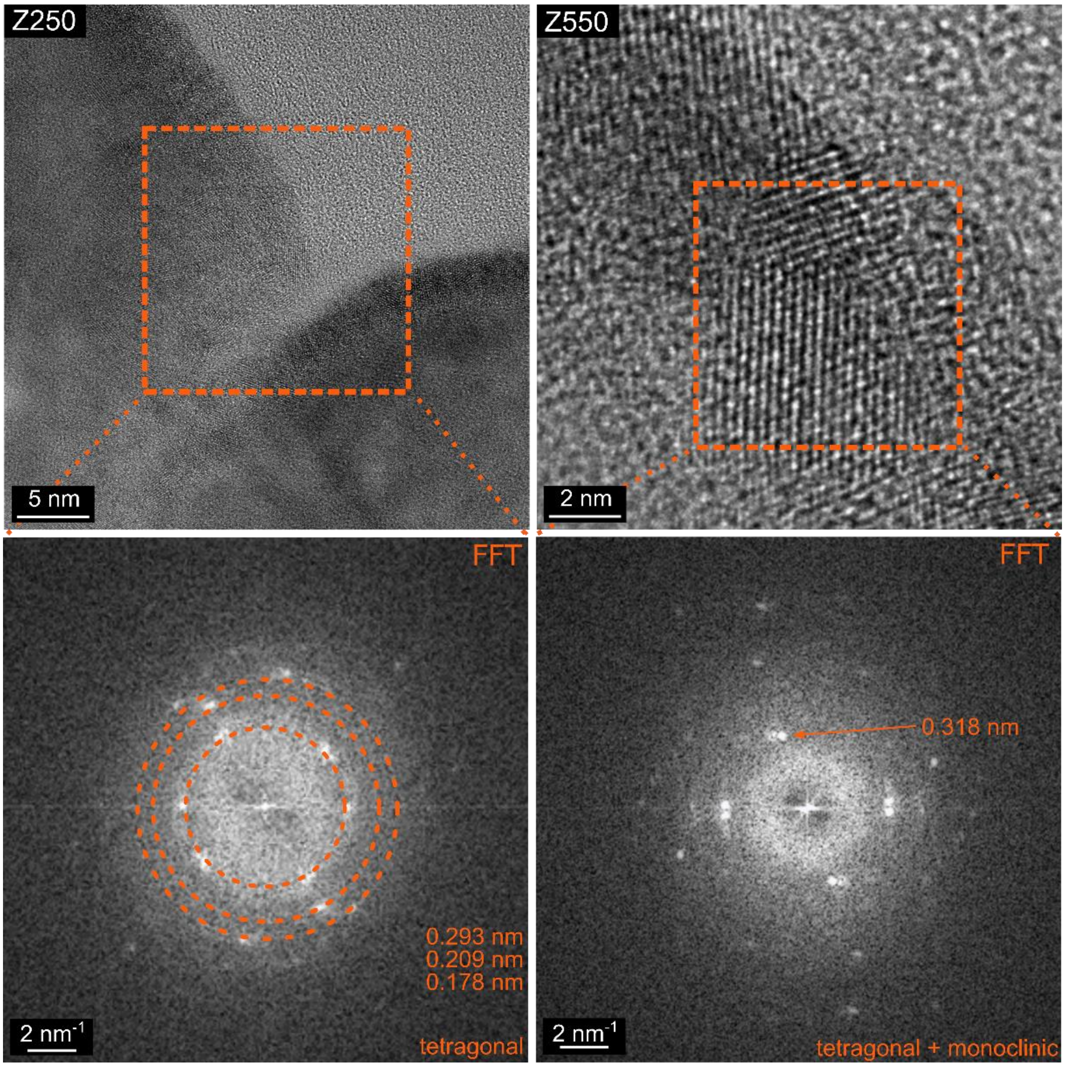

Figure S5. TEM images of FIB-prepared particle cross-sections of Z250 and Z550 and FFT patterns of the highlighted areas.

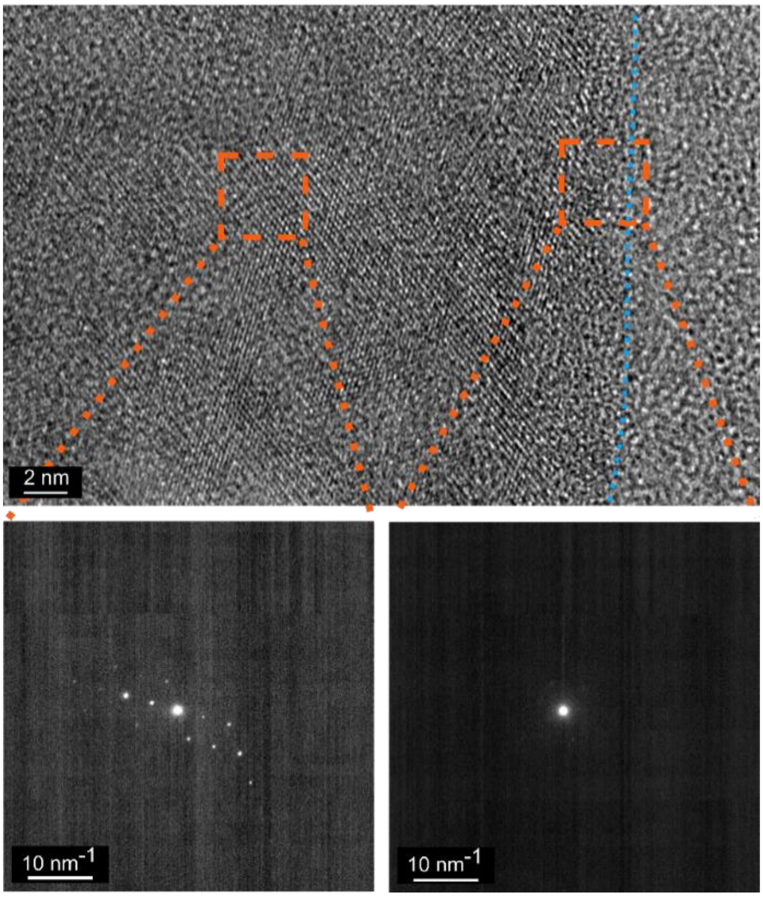

Figure S6. TEM image of a FIB-prepared particle cross-section of Z700 with NBED patterns collected from the surface and the “bulk". The blue dotted line indicates the boundary between NCM85 particle and FIB-deposited layer applied during sample preparation.

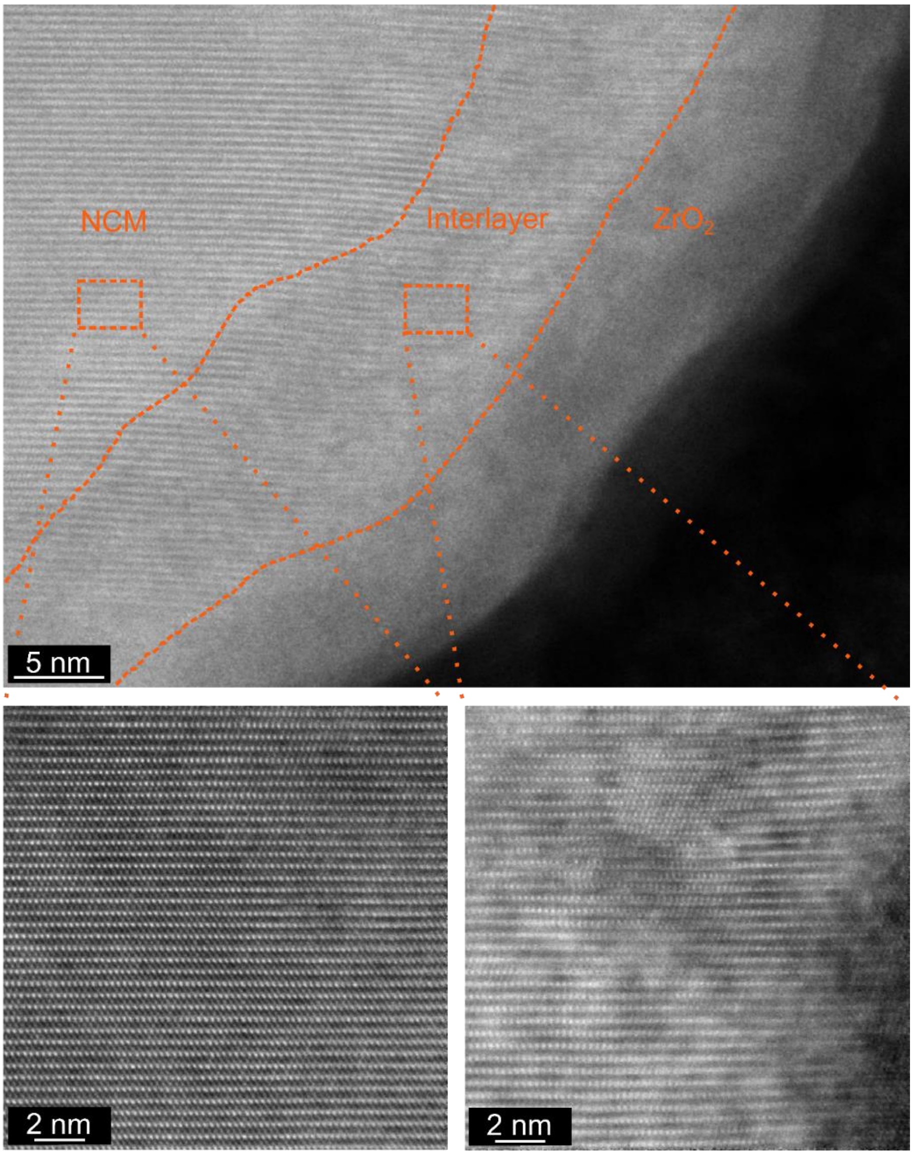

Figure S7. High-resolution STEM images of the Z700 particle surface, with dashed lines indicating the presence of an interlayer (between NCM85 and ZrO_2 coating). Dislocations are apparent in the interlayer.

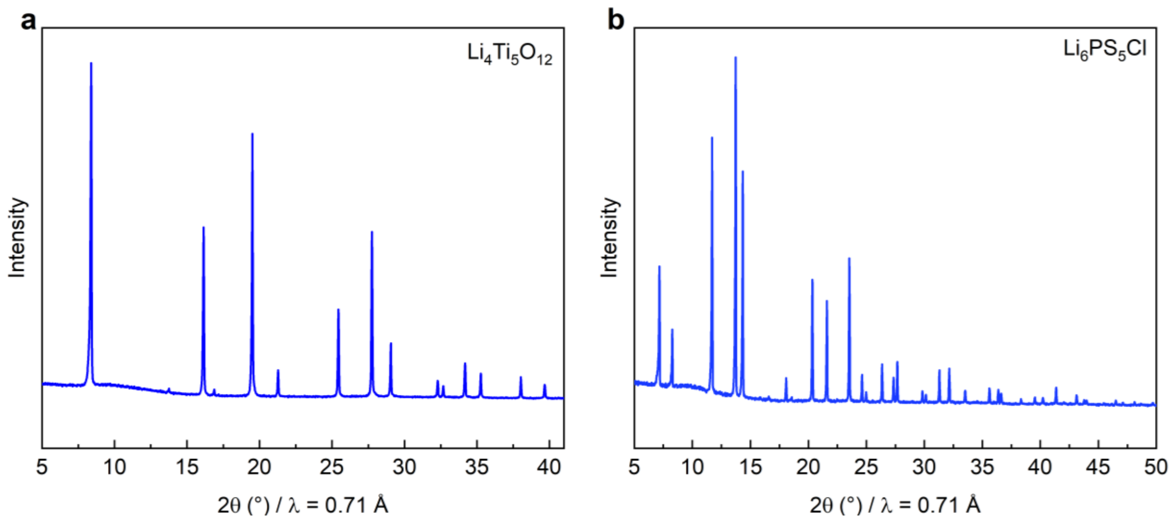

Figure S8. XRD patterns of (a) the anode material $Li_4Ti_5O_{12}$ and (b) the solid electrolyte Li_6PS_5Cl used for SSB cell testing in this study.

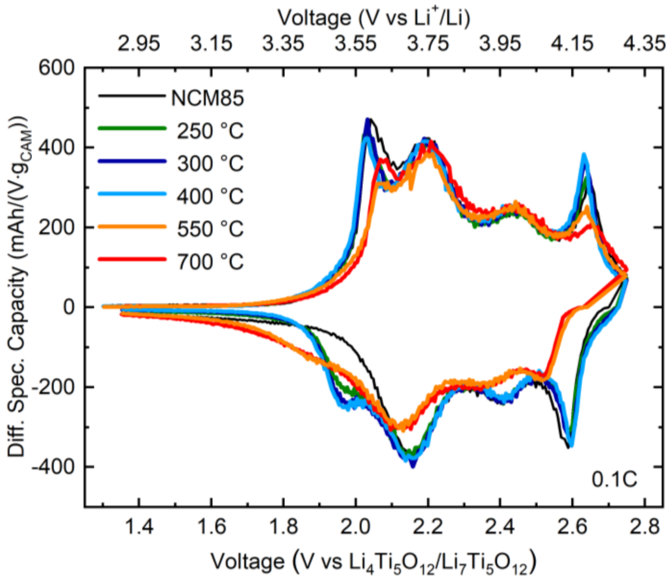

Figure S9. First-cycle differential capacity plots of the uncoated NCM85 and ZrO_2@NCM85 CAMs in SSB cells cycled at 0.1C and 45 °C in the voltage range 2.9-4.3 V vs Li^+/Li.

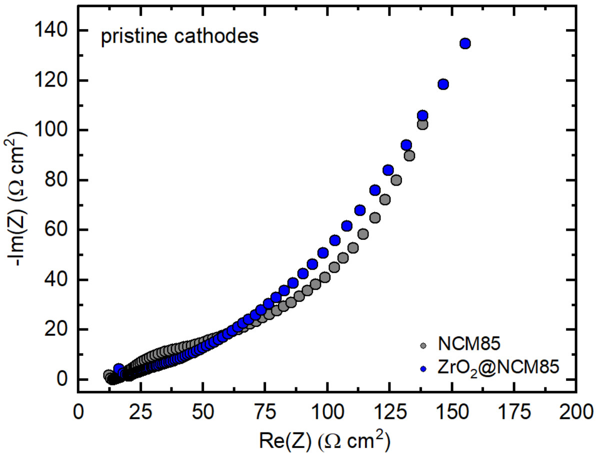

Figure S10. Nyquist plots of the electrochemical impedance of representative SSB cells in the pristine state at 45 °C.

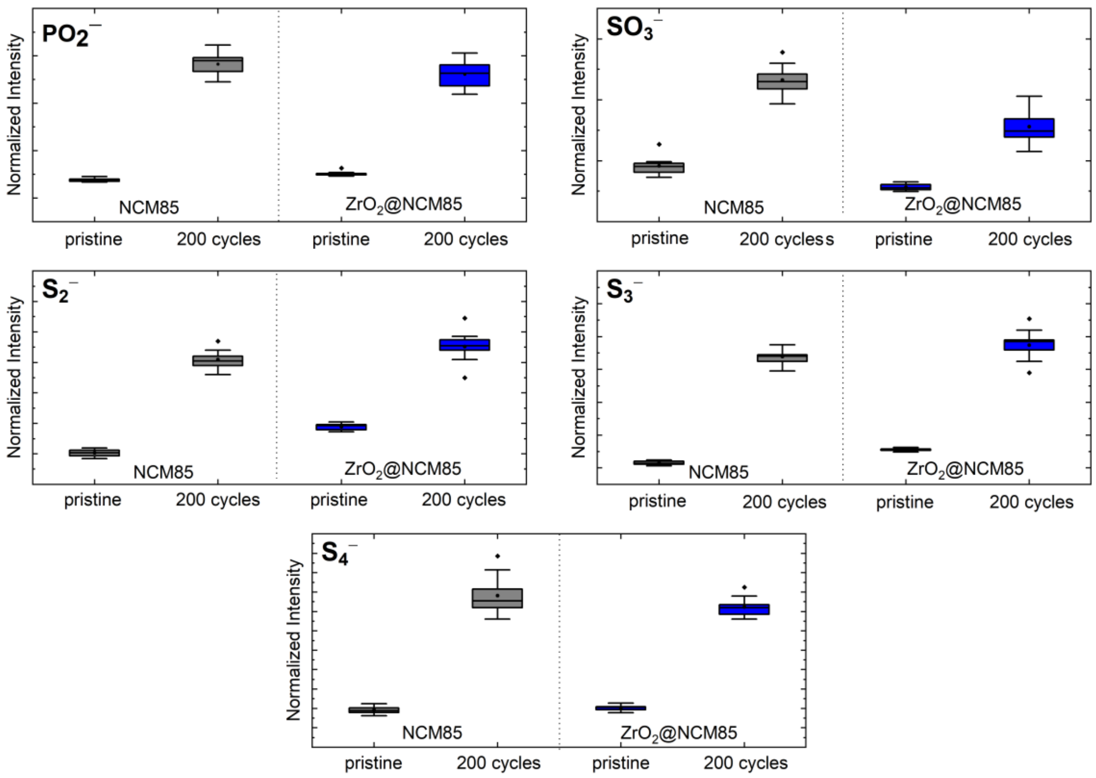

Figure S11. Box plots of measured intensities for species detected in the cycled cathodes by ToF-SIMS.

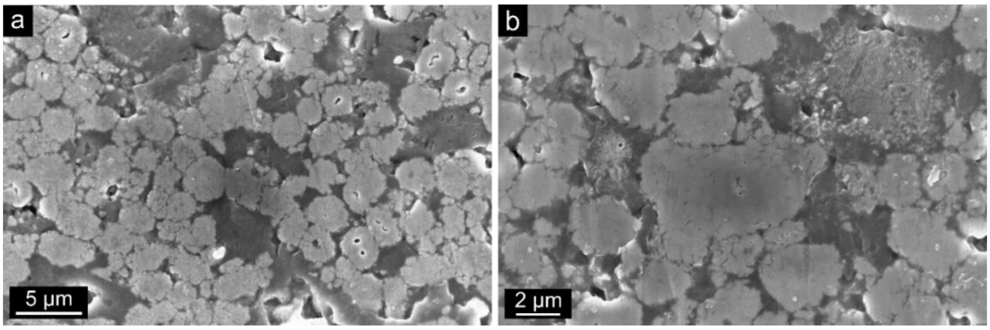

Figure S12. SEM images of pristine cathode cross-sections containing the uncoated NCM85 (prepared by ion milling).

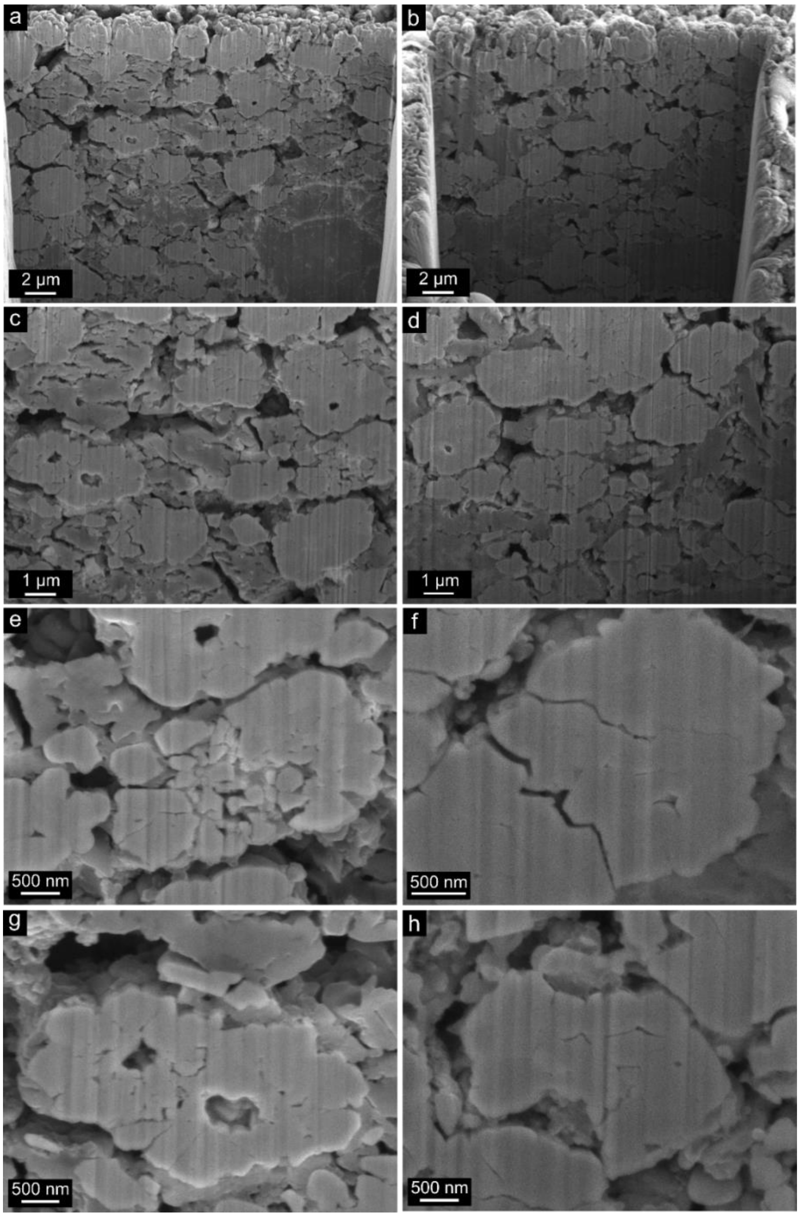

Figure S13. SEM images of FIB-prepared cross-sections of cathodes containing (left column) the uncoated NCM85 and (right column) ZrO_2@NCM85 after 200 cycles at 0.5C and 45 °C.

Estimating the diffusion coefficient for zirconium ions in NCM85 at 700 °C

The diffusion coefficient can be estimated from the ion-concentration profile based on the STEM-EDS data (see below) after annealing for 30 min. The highest Zr^{4+} concentration was set as zero point, from which the scanning distance was derived. A constant background was subtracted based on the average concentration beyond 50 nm. The diffusion coefficient can be determined using the Matano method. The Matano plane is drawn such that the areas A1 and A2 are equal.

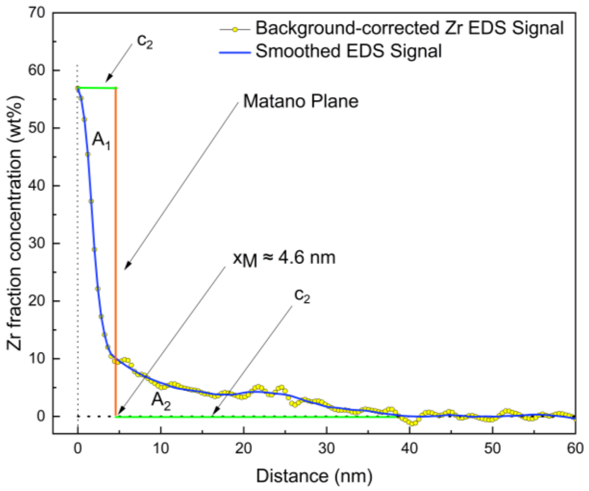

Zr^{4+} concentration profile after annealing at 700 °C for 30 min with denoted Matano plane.

Assuming the ideal case of two infinite half spaces, equation 1 is a solution to Fick's second law.

$$c(\mathrm{x},\mathrm{t}) = c_1 + \frac{c_2 - c_1}{2} \cdot \left(1 - \mathrm{erf}\left(\frac{x}{2\sqrt{D \cdot t}}\right)\right), \quad \text{(Eq. 1)}$$

where c_2 and c_1 are the ion concentrations at the surface and in the bulk of the NCM85, respectively, x represents the distance from the Matano plane and erf is the error function with:

$$\mathrm{erf}(\mathrm{z}) = \frac{2}{\sqrt{\pi}} \int_0^{\mathrm{z}} \mathrm{e}^{-t^2}\, \mathrm{d}t. \qquad \text{(Eq. 2)}$$

The formula is applied for the distances 15, 20, 25, 30, 35 and 40 nm from the surface and the average diffusion coefficient is calculated to be $D \approx 3.8 \cdot 10^{-16}\ \frac{\mathrm{cm}^2}{\mathrm{s}}$.
